# Human Guide Training to Improve Hospital Accessibility for Patients Who Are Blind: Needs Assessment and Pilot Process Evaluation

**DOI:** 10.2196/64666

**Published:** 2025-07-03

**Authors:** Tyler G James, Sarah Hughes, Christa Moran, Sherry Day, Michael M McKee

**Affiliations:** 1 Department of Family Medicine and Center for Disability Health and Wellness, University of Michigan, 1018 Fuller St., Ann Arbor, MI, 48104, United States, 1 734-998-4797; 2 ASL Interpreter Services, Office of Patient Experience, Michigan Medicine, Ann Arbor, MI, United States; 3 Department of Ophthalmology and Visual Sciences, University of Michigan, Ann Arbor, MI, United States

**Keywords:** low vision, wayfinding, human guide, accessibility, health services, patient education, rehabilitation, web-based training, visual impairment, orientation and mobility

## Abstract

**Background:**

People with disabilities are a priority population for health services research. People who are blind or have low vision (B/LV) are a segment of this priority population, who experience difficulty in accessing health care facilities due to architectural and navigational barriers. These barriers persist despite disability civil rights law in the United States.

**Objective:**

The purpose of this study is to report on a program that was developed to train way finders in human guide technique for people who are B/LV.

**Methods:**

This study took place at Michigan Medicine, an academic medical center in southeast Michigan. We conducted a needs assessment through cohort discovery and soliciting expert feedback. The human guide training program was developed using the PRECEDE-PROCEED health promotion program development model and targeted health care volunteers and staff. The intended components included in-person training, a web-based module, and tip sheets. Due to COVID-19, the in-person training was not implemented. We report findings from a process evaluation, measuring reach, knowledge, behavioral capability, and satisfaction pre- and postprogram.

**Results:**

In total, 87 participants completed the training, and most of them were Michigan Medicine volunteers. There were significant improvements in behavioral capability related to the human guide technique. Participants were satisfied with the training and provided recommendations for more detailed demonstrations and scenarios in future training sessions.

**Conclusions:**

The training improves participants’ knowledge and confidence in providing wayfinding assistance to patients who are B/LV. However, further in-person training is recommended to provide hands-on experience and detailed feedback. Addressing architectural barriers and providing accessible patient education materials is crucial for improving health care accessibility for patients who are B/LV.

## Introduction

### Background

Over 12.8 million adults in the United States report vision difficulty (ie, blind/low vision [B/LV]) [1]. This number is projected to increase as the population ages, in addition to an increase in chronic conditions that lead to being B/LV. For example, recent estimates indicate that the number of people who are legally B/LV over the age of 40 years in the United States is expected to double to more than 8 million by 2050, and another 16.4 million are expected to have difficulty seeing with correctable vision loss [2]. People who are B/LV are more likely than those who are not to have chronic conditions including diabetes, heart disease, hypertension, injuries, depression, and premature death [3-8]. The etiologies of B/LV are numerous and vary widely ranging from genetic, congenital, or acquired conditions, and may be stable or progressive. Patients who are B/LV require a comprehensive care coordination approach, which includes accessible health care facilities and health care providers with B/LV expertise.

Disability civil rights law in the United States mandates accessible health care environments for people with disabilities, including people who are B/LV. The primary legislation addressing accessibility in health care settings is the Americans With Disabilities Act (ADA) of 1990 [9]. The ADA prohibits discrimination against individuals with disabilities in all areas of public life, including employment, transportation, public accommodations, and access to state and local government services. Regarding health care specifically, the ADA requires health care providers, facilities, and programs to ensure equal access for individuals with disabilities. This includes making reasonable modifications to policies, practices, and procedures to accommodate the needs of people with disabilities, providing effective communication, and removing architectural and other barriers that may prevent access. In addition to the ADA, other laws and regulations may also impact accessibility in health care settings, such as Section 504 of the Rehabilitation Act of 1973 and the Patient Protection and Affordable Care Act (ACA) of 2010. These laws further emphasize the importance of ensuring equal access to health care for individuals with disabilities. More recently, the ACA’s Section 5307 mandates the U.S. Department of Health and Human Services to collaborate with external organizations to develop, evaluate, and disseminate curricula to address “cultural competency, prevention, public health proficiency, reducing health disparities, and aptitude for working with individuals with disabilities” [10].

Despite these mandates, patients who are B/LV experience disability-related accessibility barriers in health care settings [11-13]. Among these include inaccessible information, lack of communication support, navigational/wayfinding challenges, inaccessible medical equipment, digital accessibility issues, and attitudinal barriers. Print materials such as forms, brochures, and instructions are often not available in accessible formats, such as braille, large print, or electronic text, making it challenging for B/LV patients to access important health care information independently. Moreover, B/LV patients may encounter challenges in communicating with health care providers if there are no provisions for alternative communication methods such as screen readers for electronic health records, tactile sign language interpreters (for people who are DeafBlind), large print materials or audio descriptions of visual information and instructions [12]. In addition, patients who are B/LV may also encounter attitudinal barriers from health care providers or staff who may have misconceptions or lack awareness (due to lack of training) about the capabilities and needs of B/LV individuals, leading to stigma, discrimination, or inadequate care [12,13].

B/LV patients often encounter various architectural barriers in health care settings that can hinder their ability to navigate independently and access health care services effectively [12,14]. Architectural features such as narrow doorways, high thresholds, stairs without handrails or tactile indicators, and uneven flooring can pose significant challenges for B/LV individuals to navigate safely. Health care facilities with complex layouts, multiple corridors, and interconnected buildings can be particularly challenging for individuals who are B/LV to navigate independently without clear signage and wayfinding cues. This can make it challenging for B/LV individuals to find their way to reception areas, exam rooms, or other health care services [14]. Addressing these navigational barriers is necessary to ensure accessibility (and compliance with disability civil rights law) and requires addressing the environment through which a B/LV patient moves.

### Orientation and Mobility for People Who Are B/LV

#### Overview of Orientation and Mobility

The process through which a person navigates indoor and outdoor environments is known as orientation and mobility. Orientation and mobility encompass a range of skills and techniques to understand one’s surroundings, move around confidently, and achieve greater independence. In the case of people who are B/LV, orientation and mobility skills can be used to compensate for reduced visual information [15]. Orientation skills focus on understanding one’s position within a given space, such as a room, building, or outdoor environment. Orientation skills help individuals create mental maps of their surroundings, including landmarks and spatial relationships, through training on auditory cues, feeling changes in terrain, and using other senses to gather information about the environment. Mobility skills focus on how individuals move safely and efficiently through different environments. This includes techniques for walking with a white cane or guide dog and using public transportation.

Orientation and mobility training teaches people who are B/LV how to problem-solve, facilitates independence when faced with challenges or unfamiliar situations, and is tailored to the individual’s needs, goals, and vision loss. These skills are particularly important in environments that are not designed explicitly for accessibility, such as a hospital. Through using orientation and mobility skills, a person who is B/LV can find alternative routes, seek assistance from others, or use technology to gather information about the environment before arriving or in real time. Despite these benefits, not all people who are B/LV receive orientation and mobility training. To our knowledge, there are no prevalence estimates of the number of B/LV people who receive orientation and mobility training, but access to this training is impacted by the type of vision condition and other disabilities, access to social services, and geographic region [16,17].

#### Human Guide

A human guide (historically called a “sighted guide”) is someone who assists a B/LV person in navigating their surroundings safely and independently. Human guides provide physical assistance and verbal guidance to help the B/LV individual move through various environments with confidence [18]. This assistance can be particularly helpful in unfamiliar or crowded settings where navigation may become challenging. Particularly, the use of human guides in health care may serve as a facilitator to patient wayfinding, ensuring B/LV patients can navigate architecturally complex and crowded hospital or clinic settings. Human guide technique (also known as “guide technique”) is a method used by sighted individuals to assist B/LV or visually impaired people in navigating their surroundings safely and independently. It involves the guide providing physical support and verbal cues to aid the B/LV individual move through various environments with confidence. Tasks and responsibilities of the human guide include but are not limited to offering an arm, providing verbal directions, describing surroundings, and assisting with obstacles. It is most important for human guides to communicate effectively with the patient who is B/LV, respecting autonomy and independence while providing assistance in a supportive and respectful manner.

### Overall Aim and Objectives

The overall aim of this study was to develop a program to improve navigational access for patients who are B/LV by developing and evaluating a human guide training for staff and volunteers at a large academic medical center in southeast Michigan. Specifically, our aims were to (1) develop and implement an in-person awareness and skill-building training for hospital staff and volunteers, teaching how to provide Human Guide technique; (2) adapt the in-person Human Guide training for electronic delivery via an asynchronous, web-based learning module; and (3) conduct a process evaluation of the in-person training and web-based (asynchronous video) learning module.

As described in the forthcoming sections, the in-person training was not implemented due to infection prevention precautions (related to COVID-19). Information on the design of the in-person training is provided to facilitate implementation by other health care organizations.

## Methods

### Setting

Michigan Medicine is an academic medical center operated by the University of Michigan Medical School. Located in southeast Michigan, Michigan Medicine operates a level 1 trauma center emergency department for adults and children, and operates an adult and children’s hospital, and specialty centers in ophthalmology, oncology, and cardiovascular medicine.

### Ethical Considerations

All activities involving human participants reported in this study were reviewed by the Medical Institutional Review Board at the University of Michigan and deemed quality improvement activities (HUM00208772). Therefore, the study was exempt from ethics review.

### Needs Assessment

A needs assessment was conducted through two methods: (1) cohort discovery and (2) feedback from subject matter experts. These methods were chosen based on recommendations from the Centers for Medicare and Medicaid Services [19,20] to identify the number of people who are B/LV and identify barriers at points of contact throughout the health system.

#### Cohort Discovery

We identified the prevalence of patients who are B/LV within Michigan Medicine through DataDirect, a cohort discovery tool managed by the University of Michigan’s Data Office for Clinical and Translational Research. DataDirect can be used for cohort discovery to estimate frequencies of patient groups based on specified criteria. For cohort discovery, we searched for the frequency of living patients who have an ICD-9-CM (International Classification of Diseases, Ninth Revision, Clinical Modification) or ICD-10-CM (International Classification of Diseases, Tenth Revision, Clinical Modification) diagnosis code indicating blindness or vision impairment.

#### Subject Matter Expert Feedback

Subject matter experts were solicited for feedback prior to the creation of the intervention. We met through the University of Michigan’s Center for Disability Health and Wellness Accessibility Task Force, a group of researchers, clinicians, and staff at the University of Michigan and Michigan Medicine dedicated to improving accessibility for patients with disabilities. Experts included the (1) ASL (American Sign Language) Interpreter Services Supervisor, (2) Patient Civil Rights Coordinator, (3) ADA Compliance Officer, and (4) a community member who has low vision. We asked for feedback on the need for a patient accessibility program for people who are B/LV, and how this program might be developed and implemented.

### Human Guide at Michigan Medicine Training Program

#### Situation and Priorities

The U.S. Centers for Medicare and Medicaid Services recommends that hospitals disseminate effective training programs to address barriers to health care accessibility for blind patients [20]. Prior to the implementation of the Human Guide at Michigan Medicine Program, there was no organization-wide training teaching skills to improve accessibility for patients who are B/LV. In addition, in mid-2021, clinic staff members reported to the Office of Patient Complaints and the Office of Patient Experience that blind patients were arriving late or missing their appointments due to wayfinding accessibility issues within Michigan Medicine facilities. This presented an immediate need for a program to be responsive to policy- and patient-level needs.

Initially, this training program was conceived as a collaboration between the Center for Disability Health and Wellness and the Office of Patient Experience, specifically the latter’s HOPE Ambassador program. The HOPE Ambassadors are a group of volunteers who serve as on-site guides, assisting patients and visitors with navigating the health care facility. Before the COVID-19 pandemic, there were approximately 160 HOPE Ambassadors across 3 facilities who spoke 13 different languages. Given the HOPE Ambassadors’ role in guiding patients and knowledge of routes within the hospitals, we determined that expanding their role to serve as Human Guides would serve the addressable need.

Concurrently with the conceptualization of this program, the Center for Disability Health and Wellness was leading the implementation of the Disability and Accommodations Tab: a standardized data collection instrument for patients to report disability-related accommodation needs through the electronic health record [21]. Navigation via a Human Guide is included in this questionnaire as a potential disability-related accommodation, and Human Guide–related skills are also applicable in the clinical setting; therefore, we decided to expand the scope of the training from HOPE Ambassadors to all Michigan Medicine staff and volunteers.

#### Program Development

Program development was led by a Master Certified Health Education Specialist (MCHES) with 9 years of experience in health promotion program planning and evaluation, and 7 years of experience working with people who are B/LV (TGJ). An MCHES is a person who has demonstrated skills and training in health promotion program planning, implementation, and evaluation [22]. The core program planning group included stakeholders from Michigan Medicine’s Center for Disability Health and Wellness (MMM, Co-Director), Michigan Medicine’s Office of Patient Experience (Quinlan Davis, HOPE Program Coordinator), Michigan Medicine’s Interpreter Services (Christa Moran, ASL Interpreter Services Supervisor), Ann Arbor Center for Independent Living (William Purves, Director of Planning and Program Development) who also provided a patient perspective, and a Certified O&M Specialist and Teacher of the Vision Impaired (Lori Board).

**Figure 1.**
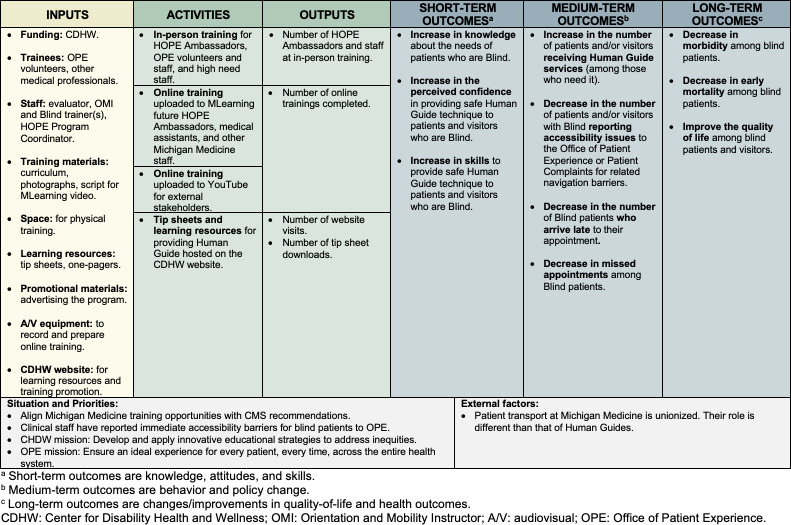
Logic model of the Human Guide at Michigan Medicine program. Yellow indicates inputs and resources, green indicates processes, and blue indicates outcomes.

We applied methods from health promotion program planning, including aspects of the PRECEDE-PROCEED model [23,24], identifying stakeholders, developing a logic model, and aligning theory with program methods [25]. Typically, the PRECEDE-PROCEED model is applied to understand quality-of-life issues among a priority population (*social diagnosis*), and then an *epidemiological assessment* is completed before identifying modifiable predisposing, enabling, and reinforcing factors (*ecological assessment*) [24]. Through this process, program planners align administrative, policy, and educational interventions with changeable factors affecting the quality-of-life of the population. Given the established priority of developing a Human Guide intervention, which would be responsive to patient- and policy-level needs, we did not conduct the preparatory phase of identifying interventions to change behavioral antecedents. Instead, we focused on a high-need intervention with high probability of implementation—increasing the capacity of existing volunteers (eg, HOPE Ambassadors) and staff to improve accessibility for B/LV patients. A logic model of the program is provided in Figure 1, and theory alignment is provided in the next section.

#### Program Components and Theory

The program had one overarching goal: increase the number of Michigan Medicine staff, volunteers, and contractors who have the skills to serve as Human Guides. To accomplish these goals, we intended to implement a three-component training program consisting of (1) in-person training, (2) a web-based, asynchronous learning module, and (3) a tip sheet or work guide. These three program components were designed to align with Social Cognitive Theory and theories of information processing (eg, the Consumer Information Processing Model) targeting behavior change constructs of behavioral capability, skills, and knowledge [25,26]. A description of how these constructs were applied in program development is provided in Table 1. As demonstrated in Table 1, the in-person training program targeted more theoretical constructs (compared to the web-based learning module) from Social Cognitive Theory related to behavioral capability (given the interactive nature of the in-person training).

**Table 1.** Theoretical alignment and application in the in-person and video-based Human Guide training program.

Outcome	Theory	Theory-based method	Training component
Knowledge of Human Guide	Theories of information processing (eg, Consumer Information Processing Model and Cognitive Load Theory)	Alignment with information processing capacity	In-person and video-based training: Teaching only the most important and useful points necessary to provide Human Guide safely and efficiently, thereby reducing information redundancy.
		Chunking	In-person and video-based training: Providing instruction on different types of O&M tools used by Blind people, followed by providing Human Guide techniques under different scenarios.
		Using imagery	In-person training: Training in a setting similar to clinical or real-world contexts. Video-based training: Using images and videos that are similar to real-world Human Guide scenarios.
Behavioral capability and skills related to Human Guide	Social Cognitive Theory	Active learning	In-person training: Alternating between didactic and interactive/hands-on simulations.
		Enactive mastery experiences	In-person training: Increasing the challenge of simulated tasks to help indicate ability.
		Feedback	In-person training: Providing direct feedback to trainees about their skills and opportunities to improve the Human Guide technique.
		Guided practice	In-person training: Giving the opportunity for multiple repetitions of providing Human Guide technique, and debriefing with facilitators and peers.
		Modeling or observational learning	In-person and video-based training: Providing examples (through demonstration) modeling Human Guide in specific scenarios.
		Verbal persuasion	In-person and video-based training: Using messages that affirm the ability of participants to provide a Human Guide.
Awareness of Human Guide training	Diffusion of Innovations Theory	Observability	Incentive for in-person and video-based training: Individuals who are not aware of the training program will observe their peers having a lapel pin and know they have adopted the training.

The initial plan was to implement a training facilitated by a Certified Orientation and Mobility Specialist and B/LV cofacilitators. The training would be focused on delivering the most needed content knowledge and provide ample time for skills-based practice through roleplaying and simulations. The training curriculum covered the following core areas: (1) exposing trainees to the navigational wayfinding challenges experienced in health care settings; (2) differentiating between different conditions that cause blindness; (3) providing an overview of orientation and mobility for people who are B/LV, including the use of guide dogs; and (4) situations and techniques for providing Human Guide. The Human Guide-specific instruction included (4a) the general set-up of the Human Guide technique, (4b) providing Human Guide outdoors, (4c) navigating doorways, (4d) walking at a comfortable pace, (4e) approaching steps and taking stairs, (4f) taking elevators, and (4g) orienting in a waiting room. Throughout the training, the curriculum underscored the importance of maintaining patient or visitor autonomy by asking their preferences or wants.

The in-person training, however, was canceled after multiple scheduling attempts due to infection prevention concerns regarding the COVID-19 pandemic, and hospital restrictions on the number of volunteers permitted. The web-based learning module was designed as a very brief, asynchronous, video-based, nonexperiential training intended to serve as a “survival” training. This format and length were chosen to accommodate the busy schedules and workflows of clinical staff in Michigan Medicine. Video content was planned to be filmed at the in-person training; however, due to the aforementioned changes, we instead filmed at a Michigan Medicine clinical location. The in-person curriculum was shortened, but the abovementioned covered-areas remained the same. The training script and imagery were reviewed by subject matter experts, including a Certified Orientation and Mobility Specialist, for accuracy prior to distribution. The final training is available on the web, for free (see Data Availability section).

#### Program Advertisement

The program was advertised through the University of Michigan’s Center for Disability Health and Wellness. The training was immediately integrated into the HOPE Ambassador program as required training prior to becoming a volunteer. Other avenues of promotion included (1) advertisement by MDisability, a family medicine clinical research program aimed at improving primary care for people with disabilities; (2) email newsletters through the Council on Disability Concerns at the University of Michigan; (3) on public-facing and internal websites; and (4) through the primary newsletter sent regularly to Michigan Medicine employees.

### Process Evaluation

This study was designed as a process evaluation using methods common in health promotion program evaluation [27]. Process evaluations are a type of formative evaluation used to assess the quality and delivery of program activities and components. Although process evaluations can include a variety of measures, most process evaluations include measures of reach (ie, how many people received the program) and early indicators if the program is working as intended (ie, early impact indicators) [27].

In our process evaluation, we included four measures: (1) reach, (2) knowledge, (3) behavioral capability, and (4) satisfaction. Reach was measured based on the number of people who accessed and completed the training. Knowledge was operationalized through a 5-item program team-developed knowledge questionnaire focused on aspects included in the training (see Table 2). Participants were asked to respond to these knowledge questions as being true, false, or don’t know. Incorrect and “don’t know” responses were recoded as incorrect. Behavioral capability, or self-reported understanding of having the skill needed to complete a task, was asked through three items focused on (1) providing clear directions, (2) providing a human guide technique, and (3) providing a human guide technique with a service animal. Satisfaction was operationalized through three questions: (1) relevance to the organization, (2) overall satisfaction, and (3) recommending to colleagues. Behavioral capability and satisfaction questions were responded to on a 5-point Likert-type scale (ie, strongly disagree, disagree, neutral, agree, and strongly agree), with a score of 5 being “strongly agree.” Individual questions are provided in the Results section.

Data were analyzed in the R statistical environment using JASP (Jeffrey’s Amazing Statistics Program). We summarize the reach and knowledge questions using frequencies and percentages. We calculated means to describe behavioral capability and satisfaction. Pre- and posttest scores for behavioral capability were assessed for change using paired samples *t* tests with Cohen *d* effect sizes.

**Table 2.** Trainee characteristics representing the reach of the human guide training program.

Characteristics	Values, n (%)
Affiliation with Michigan Medicine	
Unaffiliated	25 (29)
Nonpatient facing staff	7 (8)
Patient facing staff	10 (11)
Volunteer	45 (52)
Previously trained^a^	24 (28)
Advertising source^a^	
Center for Disability Health and Wellness	12 (14)
Office of Patient Experience	24 (28)
Recommended by a friend or colleague	24 (28)
Web search	8 (9)
ADA^b^ technical assistance network	5 (6)
Other University of Michigan group	13 (15)

a One case missing (ie, analytic n=86).

b ADA: Americans With Disabilities Act.

## Results

### Needs Assessment

There are inadequate published definitions of diagnostic codes related to capturing patients who are B/LV. We used ICD-9-CM and ICD-10-CM diagnostic codes used by Ratakonda and colleagues [28] when studying potentially preventable hospitalizations among people with sensory disabilities. We identified that there were 55,159 B/LV patients who had an encounter in Michigan Medicine during the 2 years prior to the program (Table 3).

Subject matter experts agreed that navigating the health care system’s clinics and hospitals was taxing on patients who are B/LV. The timeliness of addressing this issue was underscored by experts as the Office of Patient Experience recently reported there had been patients arriving late to appointments, despite having been on the premises before the appointment start-time, due to challenges visually navigating the environment. In these cases, not all patients were able to be worked back into the clinic’s schedule leading to a delay in receiving care. In one instance, a complaint was escalated and social workers became involved to better support the patient.

**Table 3.** Patients with problem list diagnoses related to blindness.^a^

High-level diagnosis	Patients encounter between December 31, 2021, and January 1, 2023, n (%)	Total patients in electronic health record, n
Neurological vision impairment (eg, visual field defect and central scotoma)	6072 (53.2)	11,412
Retina	12,739 (50.2)	25,380
Age-related macular degeneration	25,977 (44.9)	57,912
Other vision impairments (eg, blindness and low vision, cortical blindness)	19,742 (54.3)	36,381

a Patients may be in more than one high-level diagnosis category.

### Process Evaluation

In total, 87 people completed the web-based learning module and were included in the process evaluation dataset. These participants took the training between February 21, 2023, and December 31, 2023. The majority of these trainees were volunteers at Michigan Medicine (45/87, 52%). Over one-fourth (25/87, 29%) of trainees were unaffiliated with Michigan Medicine or the University of Michigan (eg, some trainees were from other states). Less than one-third of participants (24/87, 28%) had previously completed a human guide training. The most common advertising sources for learning about the training were from the Office of Patient Experience (24/87, 28%) and recommendations from friends or colleagues (24/87, 28%).

**Figure 2.**
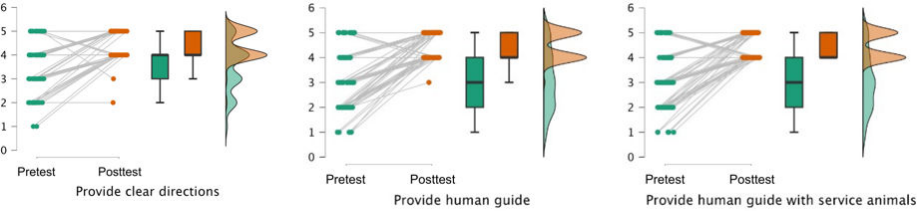
Rainfall plots of pre- and posttraining behavioral capability as reported by human guide trainees. Green color indicates pretest scores and distributions, while orange color indicates posttest scores and distributions.

Completing the human guide training required scoring a 100% on the 5-item knowledge quiz; therefore, we did not calculate pre- and posttest score changes. At pretest, however, the most difficult question was “If a person identifies as blind, it is appropriate to call the person vision impaired” with almost 65% (55/85) of participants scoring incorrectly (see Table 4). This contrasted to knowledge related to not making noises to distract service dogs (correct: 79/84, 94%). There was a significant increase in self-reported behavioral capability of (1) providing clear directions, (2) providing a human guide (without a service dog), and (3) providing a human guide with a service dog. Effect sizes of the mean differences were high (*d*s>0.9) but expected given the immediate posttest nature of the survey. Rainfall plots of pre- and posttraining behavioral capability show that while there was large variability in pretest responses, the responses at posttest clustered around 4 (“agree”) and 5 (“strongly agree”; see Figure 2).

Trainees reported being satisfied with the training (mean 4.5, SD 0.57) and would recommend it to their colleagues.

**Table 4.** Pre- and posttraining knowledge, behavioral capability, and satisfaction reported by human guide trainees.

Domain	Score
Knowledge (pretest), n/N (%)	
People with blindness cannot see at all. [False]	78/86 (91)
There is a legal definition of blindness and low vision. [True]	74/84 (88)
Age-related macular degeneration is a common cause of vision loss. [True]	71/85 (84)
If a person identifies as blind, it is appropriate to call the person vision impaired. [False]	30/85 (35)
When I see a service dog, I am permitted to make noises that may distract the dog as long as I do not touch the dog. [False]	79/84 (94)
Behavioral capability	
I can provide clear directions to patients/visitors who are blind/low vision.	
Pretest, mean (SD)	3.48 (1.04)
Posttest, mean (SD)	4.38 (0.60)
Cohen *d* (95% CI)	0.93 (0.64‐1.21)
I can provide Human Guide technique to patients/visitors who are blind/low vision.	
Pretest, mean (SD)	3.12 (1.20)
Posttest, mean (SD)	4.50 (0.53)
Cohen *d* (95% CI)	1.09 (0.79‐1.38)
I can provide Human Guide technique to patients/visitors who are blind/low vision, and who use service animals.	
Pretest, mean (SD)	3.07 (1.16)
Posttest, mean (SD)	4.50 (0.50)
Cohen *d* (95% CI)	1.23 (0.91‐1.54)
Satisfaction (posttest), mean (SD)	
This training is relevant to providing patient care at Michigan Medicine.	4.56 (0.62)
Overall, I was satisfied with this presentation.	4.50 (0.57)
I would recommend this training to my coworkers.	4.58 (0.53)

### Recommendations for Improvement

Participants were asked to provide feedback on how to improve the web-based learning module; 16 participants provided free-text comments. Although some of these comments were positive, most responses provided critical feedback about trainees’ expectations and needs. These needs were well aligned with the goals of the in-person training, including more demonstration, enactive mastery experiences, and simulation in clinical settings.

*I was a little confused about how to offer your hand and some other minor details about guiding, so a full video scenario would be helpful and just many videos of watching guides interact with patients over and over would help too*.

Trainees also indicated concern with navigating through doorways.

*I would only recommend extending the camera view of walking through doorways. This is a hard skill and I was hopeful to have more of a view from both sides about how to best navigate in this area.*

Other comments indicated that the training was “informative, but also rather general.” This trainee further indicated that additional practice would be needed for proficiency. Other trainees commented that they wanted to be asked additional knowledge questions related to human guide to ensure they understood the content. Lastly, three comments focused on platform or technology issues, including the training platform not allowing full screen for the videos and also a captioning error which was fixed.

## Discussion

### Evaluation Findings

Patients who are B/LV face obstacles in physically navigating health care environments, particularly complex and maze-like hospitals. These obstacles serve as barriers to effectively and efficiently engaging in health care and may implicate health care systems due to regulatory requirements with the ADA and ACA. Therefore, it is necessary to improve accessibility to effectively navigate health care facilities. The aim of this study was to develop a program to improve navigational access for patients who are B/LV by developing and evaluating a Human Guide training for staff and volunteers at a large academic medical center. Specifically, we aimed to (1) develop an in-person training, (2) adapt the in-person training for electronic delivery as a web-based learning module, and (3) conduct a process evaluation of both the in-person training and web-based learning module. As described earlier, we only accomplished Aim 2 and Aim 3. This web-based learning module focused on teaching human guide techniques to improve wayfinding assistance for people who are B/LV. This training was aligned with priorities set by the U.S. Centers for Medicare and Medicaid Services and needs in the local setting—particularly given the priorities to address poor patient experiences due to navigational barriers.

This early process evaluation suggests that the training is accurately targeting the intended skills and knowledge related to human guide technique. This training has also been adopted by the HOPE Ambassador program, improving the skillset of these wayfinding volunteers to meet the needs of people who are B/LV. The program could be improved, however. Results from the evaluation, and the early theory of change from the program, suggest the importance of providing in-person training allowing learners to practice human guide technique and receive just-in-time feedback. This would improve learners’ experience and also aid in cementing knowledge and behavior change.

Improving wayfinding assistance through human guide does not address the root cause of navigational inaccessibility in health care settings. Addressing architectural barriers requires a proactive approach to accessibility in health care facility design and renovation, as well as ongoing efforts to improve wayfinding and navigation assistance for people who are B/LV. This may involve incorporating universal design principles, installing tactile signage and markings, ensuring clear pathways and accessible entrances, providing adequate lighting, training staff to assist patients and visitors who are B/LV, and providing web-based audio descriptions or tactile maps [29,30]. Some additional recommendations are provided in Textbox 1. By removing architectural barriers, health care facilities can create environments that are more inclusive and accessible for people with disabilities, including those who are B/LV.

Textbox 1. **Recommendations to address barriers to inaccessibility in healthcare settings.**

- Address architectural barriers including stairs without handrails, lack of tactile indicators and high contrast visual indicators, and narrow doorways.
- Provide educational materials in accessible and alternative formats (eg, large print and Braille).
- Expand medical education and training to include disability competencies.
- Improve accessibility and referral to orientation and mobility specialists.
- Install tactile nurse call buttons and audible entry alarms in patient rooms.
- Ensure physical and electronic signage is in large print, high contrast, and low glare.
- Design spaces with attention to lighting, windows, and flooring and the amount of glare.
- Facilitate wayfinding through use of floor guides with visual and tactile indicators (eg, follow the black line to get to radiology), audible alerts in elevators and doorways, and the use of mobile GPS.
- Enable website and portal customization options (eg, increasing font size and contrast).
- Work with patients and visitors who are blind/low vision to identify and address accessibility concerns.

### Limitations and Recommendations for Future Programs

There are several limitations to our program and program evaluation. The lack of full program implementation due to infection control policies led to potentially missed opportunities to provide just-in-time feedback to trainees on how to improve their human guide technique. This would have been responsive to trainee’s needs, as indicated by the open-ended feedback. Despite this, the web-based learning module was liked by participants and had an associated increase in perceived behavioral capability. Still, we lack medium- and long-term outcome measurement. Collecting medium-term outcomes was too burdensome for this resource-limited training, particularly given the low incidence of patients and visitors needing human guide. In addition, the immediate pretest and posttest design is known to inflate effect size measures. To address these limitations, we recommend that future human guide trainings consider hosting an in-person training, in addition to web-based videos, and also consider longer posttest observations. The evaluation of this training could also be improved by reducing reliance on self-reported knowledge and behavioral capability, and instead use an observation checklist to evaluate trainees’ skills. Orientation and mobility specialists may be particularly well-suited to evaluating trainee skills. This could serve a dual purpose as a data source and a feedback mechanism.

### Conclusions

Patients who are B/LV experience barriers to physically navigating health care environments. Human guide is a technique that can be used to assist blind patients navigating the environment. Participants who completed our very brief, web-based video learning module reported improvements in behavioral capability with providing human guide technique to patients who are blind. We recommend the implementation of disability competency trainings in health provider preparation programs, and as required trainings for staff and volunteers working in clinical settings.

## Abbreviations

ACA: Patient Protection and Affordable Care Act
ADA: Americans With Disabilities Act
ASL: American Sign Language
B/LV: blind/low vision
HOPE: Helping Our Patients Everyday
ICD-10-CM: International Classification of Diseases, Tenth Revision, Clinical Modification
ICD-9-CM: International Classification of Diseases, Ninth Revision, Clinical Modification
JASP: Jeffrey’s Amazing Statistics Program
MCHES: Master Certified Health Education Specialist

## References

[1] Varadaraj V, Deal JA, Campanile J, Reed NS, Swenor BK (2021). National prevalence of disability and disability types among adults in the US, 2019. JAMA Netw Open.

[2] Varma R, Vajaranant TS, Burkemper B (2016). Visual impairment and blindness in adults in the United States: demographic and geographic variations from 2015 to 2050. JAMA Ophthalmol.

[3] Jones GC, Rovner BW, Crews JE, Danielson ML (2009). Effects of depressive symptoms on health behavior practices among older adults with vision loss. Rehabil Psychol.

[4] Crews JE, Chou CF, Sekar S, Saaddine JB (2017). The prevalence of chronic conditions and poor health among people with and without vision impairment, aged ≥65 years, 2010-2014. Am J Ophthalmol.

[5] Swenor BK, Wang J, Varadaraj V (2019). Vision impairment and cognitive outcomes in older adults: the Health ABC study. J Gerontol A Biol Sci Med Sci.

[6] Patel N, Stagg BC, Swenor BK, Zhou Y, Talwar N, Ehrlich JR (2020). Association of co-occurring dementia and self-reported visual impairment with activity limitations in older adults. JAMA Ophthalmol.

[7] Varadaraj V, Munoz B, Deal JA (2021). Association of vision impairment with cognitive decline across multiple domains in older adults. JAMA Netw Open.

[8] Centers for Disease Control and Prevention (CDC) (2001). Prevalence of disabilities and associated health conditions among adults—United States, 1999. MMWR Morb Mortal Wkly Rep.

[9] (1991). Americans with disabilities act of 1990. https://www.congress.gov/bill/101st-congress/senate-bill/933.

[10] (2010). Section 5307 of the patient protection and affordable care act of 2010. https://www.congress.gov/111/plaws/publ148/PLAW-111publ148.pdf.

[11] Spencer C, Frick K, Gower EW, Kempen JH, Wolff JL (2009). Disparities in access to medical care for individuals with vision impairment. Ophthalmic Epidemiol.

[12] Iezzoni LI, Rao SR, Ressalam J, Bolcic-Jankovic D, Campbell EG (2022). Incidence of accommodations for patients with significant vision limitations in physicians’ offices in the US. JAMA Ophthalmol.

[13] Heydarian NM, Hughes AS, Morera OF, Bangert AS, Frederick AH (2021). Perspectives of interactions with healthcare providers among patients who are blind. J Blind Innov Res.

[14] Ulldemolins AR, Lansingh VC, Valencia LG, Carter MJ, Eckert KA (2012). Social inequalities in blindness and visual impairment: a review of social determinants. Indian J Ophthalmol.

[15] Jacobson WH (1993). The Art and Science of Teaching Orientation and Mobility to Persons With Visual Impairments.

[16] Lam N, Leat SJ (2013). Barriers to accessing low-vision care: the patient’s perspective. Can J Ophthalmol.

[17] Kaldenberg J (2019). Low vision rehabilitation services: perceived barriers and facilitators to access for older adults with visual impairment. British Journal of Occupational Therapy.

[18] Novi R Orientation and mobility for sight deficients.

[19] (2020). Improving health care for adults with disabilities: an overview of federal data sources. https://www.cms.gov/files/document/federaldatadisability508.pdf.

[20] (2020). Improving communication access for individuals who are blind or have low vision. https://www.cms.gov/files/document/omh-visual-sensory-disabilities-brochure-508c.pdf.

[21] Halkides H, James TG, McKee MM, Meade MA, Moran C, Park S (2022). Spotlighting disability in a major electronic health record: Michigan medicine’s disability and accommodations tab. JMIR Form Res.

[22] Knowlden AP, Cottrell RR, Henderson J (2020). Health education specialist practice analysis II 2020: processes and outcomes. Health Educ Behav.

[23] Green LW, Kreuter M (2005). Health Program Planning: An Educational and Ecological Approach.

[24] Green LW, Gielen AC, Ottoson JM, Peterson DV, Kreuter MW (2022). Health Program Planning, Implementation, and Evaluation: Creating Behavioral, Environmental, and Policy Change.

[25] Bartholomew Eldredge LK, Markham CM, Ruiter RA, Kok G, Parcel GS (2016). Planning Health Promotion Programs: An Intervention Mapping Approach.

[26] Glanz K, Rimer BK, Viswanath K (2015). Health Behavior: Theory, Research, and Practice.

[27] Sharma M, Petosa RL (2012). Measurement and Evaluation for Health Educators.

[28] Ratakonda S, Lin P, Kamdar N, Meade M, McKee M, Mahmoudi E (2023). Potentially preventable hospitalization among adults with hearing, vision, and dual sensory loss: a case and control study. Mayo Clin Proc Innov Qual Outcomes.

[29] Khan S, Nazir S, Khan HU (2021). Analysis of navigation assistants for blind and visually impaired people: a systematic review. IEEE Access.

[30] Williams MA, Dubin B, Amaefule C (2016). Designing Around People: CWUAAT 2016.

[31] Resources and accommodations. University of Michigan Medical School.

